# Effectiveness of Doctor Clerks Supporting Physicians’ Work in Japan: A Systematic Review

**DOI:** 10.7759/cureus.53407

**Published:** 2024-02-01

**Authors:** Ryuichi Ohta, Miyuki Yawata, Chiaki Sano

**Affiliations:** 1 Community Care, Unnan City Hospital, Unnan, JPN; 2 Family Medicine, Unnan City Hospital, Unnan, JPN; 3 Community Medicine Management, Shimane University Faculty of Medicine, Izumo, JPN

**Keywords:** community hospital, family medicine, japan, task shifting, workload, administrative personal, healthcare quality, physician’s role, doctor clerk

## Abstract

The burgeoning administrative workload on physicians in Japan's healthcare system has necessitated innovative approaches to optimize clinical care. Integrating doctor clerks, tasked with administrative and clerical duties, has emerged as a potential solution to alleviate this burden. This systematic review aims to evaluate the effectiveness of doctor clerks in improving physicians' working conditions and patient care quality. A comprehensive literature search was conducted using Ichushi Web and Google Scholar from January 2000 to September 2023. Data were extracted on publication year, study setting, department focus, work scope, and outcomes of doctor clerk implementation. The search identified 3570 studies, with 17 meeting the inclusion criteria. Most studies were performed in general hospitals with 76.5% (13/17). The studies regarding university hospitals were 17.6% (3/17). Only one study was performed in a community hospital with 5.9% (1/17). More than half of doctor clerks worked not explicitly allocated to one department and did their work not specific to one department with 52.9% (9/17). Three studies report that doctor clerks collaborate with orthopedic surgeons. Two studies report that doctor clerks collaborate with emergency medicine physicians. Each study reports that doctor clerks collaborate with respiratory or general medicine. The most frequent is document support, with 94.1% (16/17). The second most frequent working content is consultation support, with 47.1% (8/17). The third most frequently working content is ordering support, with 23.5% (4/17). Call response, secretary work, education support, research support, conference support, and other professional support are included, each with 5.9% (1/17). Regarding clinical outcomes, five studies assessed a reduction in physician paperwork time (29.4%). Four studies assessed the frequency of the contents of doctor clerks’ work (23.5%). Four studies assessed the positive perception of physicians (23.5%). Four studies assessed the amount of the reduction in physicians’ overtime work (23.5%). Three studies assess the amount of the reduction in hospital costs (17.6%). One study assessed part-time physicians' fatigue reduction (5.9%). Each study assessed the quality of patient care, such as doctor's clerk education for standardization, increase in the number of patients accepted, reduction in medical incidents, decrease in patient waiting time, and primary to tertiary prevention. Introducing doctor clerks in Japan's healthcare system shows promise in enhancing physicians' working conditions and potentially improving patient care. However, conclusive evidence on the impact on patient care quality necessitates further investigation, serving as a foundation for future policy and healthcare system optimization.

## Introduction and background

In the dynamic healthcare field, the equilibrium between clinical responsibilities and administrative tasks has become increasingly critical, especially within Japan's healthcare framework [1]. Several strategies have been implemented to alleviate the workload of physicians, among which the adoption of doctor clerks stands out as a critical initiative [2]. The introduction of doctor clerks aims to address the growing burden of administrative duties and non-clinical tasks on medical professionals [2]. This approach goes beyond administrative efficiency; it is part of a larger strategy to reform the working conditions of doctors, thereby elevating the standard of patient care in hospitals [3]. Nevertheless, there is a notable absence of concrete evidence regarding the actual effect of doctor clerks on the enhancement of patient care quality, highlighting the necessity for a thorough investigation into their function and efficacy within Japan's healthcare system.

The role of doctor clerks is vital in redistributing the workload of physicians. Medical assistants have been employed to lessen the strain on medical doctors, resulting in decreased stress and heightened job satisfaction for physicians in hospitals and clinics [4-6]. In 2007, Japan's Ministry of Health, Labor, and Welfare advocated separating duties between doctors and other medical and clerical personnel [2]. Subsequently, in 2008, the position of doctor clerk was formally introduced in Japan as a medical assistant to lessen doctors' workload. These clerks undertake administrative tasks like document preparation and other traditionally managed by physicians but which can be delegated [2]. Their presence is intended to alleviate the demands on practicing doctors, allowing them to focus more on clinical care [7]. The scope of a doctor clerk's responsibilities varies with each healthcare facility, depending on the specific needs of the physician's workload [2,7]. Since this initiative, community hospitals in Japan have been employing doctor clerks to facilitate task redistribution among physicians. These clerks now handle a broad range of clerical tasks and are also involved in entering medical information into records following physicians' decisions. As task shifting evolves in Japan, the skills required for doctor clerks continue to grow, encompassing tasks like ordering medical tests, updating electronic medical records, preparing discharge summaries, and undertaking other administrative duties that improve healthcare quality [2,7].

Despite the strategic deployment of doctor clerks, there is a lack of empirical data substantiating the claim that they significantly enhance patient care quality [8]. The issue is dual-faceted: first, the actual impact of doctor clerks on reducing the workload of physicians needs both quantitative and qualitative assessment; second, the relationship between this workload reduction and the enhancement of patient care remains hypothetical in the absence of robust, research-based evidence. This knowledge gap forms a substantial obstacle to the informed implementation and refinement of the doctor clerk role in medical settings [9].

Considering this gap in existing research, the guiding question for this study is: "How does the integration of doctor clerks affect the quality of patient care in Japanese hospitals?" The objectives of this research are threefold: to aggregate and scrutinize the available Japanese literature on doctor clerks, evaluating their role and effectiveness in the healthcare system; to empirically determine if their activities have a positive impact on patient care quality; and to contribute to the scholarly discussion on optimizing work systems in healthcare. This study seeks to bridge the existing knowledge void, offering insights for policy and operational strategies regarding integrating non-clinical staff in healthcare environments, with the ultimate goal of enhancing patient care outcomes in Japan.

## Review

Method

Systematic Review

This systematic review was prepared according to the preferred reporting items for systematic reviews and meta-analyses (PRISMA) guidelines [10].

Data Sources

We searched for Japanese reports of working doctor clerks on Ichushi Web and Google Scholar from January 2000 to September 2023. The words used in the search were “Ishizimu Hozyosya,” “Ishi Clerk,” “Iryokei Clerk,” and “Iryo Hosa Gakari,” all translated to “doctor clerk” in English. 

Study Selection

The inclusion and exclusion criteria are listed in Table 1.

**Table 1:** Study inclusion and exclusion criteria

Criteria	Inclusion	Exclusion
Population	Doctor clerks, physicians, patients	Other healthcare professionals (nurses, pharmacists, dentists, rehabilitators, care managers)
Intervention	Doctor clerks’ assistance in physicians' work	Clinical experience only in healthcare facilities
Type of study	Qualitative, quantitative, mixed method	Non-empirical studies (editorials, news)
Other	Abstract available, year of publication >2000, conducted in Japan, including the outcomes in medical care, full text available in English or Japanese	Abstract not available, full text not available in English or Japanese

We included original papers executed in Japan that describe doctor clerk’s work effectiveness. We excluded reports of other professionals, non-empirical studies, and studies with abstract and full text not available. Results obtained in the Google Scholar search that overlapped with studies identified in the Ichushi Web search were excluded to avoid duplication.

Data Extraction

We collected and analyzed the publication year, title, working setting, charge department, study design, working contents, familial history of bleeding, and outcome of the implementation of doctor clerks.

The setting was categorized into three categories: university hospitals, general hospitals, and community hospitals, based on their affiliations and functions. University hospitals are affiliated with medical universities. General hospitals function as specific function hospitals having advanced care for patients. Community hospitals serve local communities and provide primary and secondary care.

The charge department was categorized into specific departments and non-specific. Working content was categorized into document supports (writing official documents that doctors are supposed to write, such as documents such as medical certificates, insurance documents, and nursing care documents), call response (alternatively response to calls from outside of hospitals), secretary work (supporting educational, research, and conference preparation), other professional supports (supporting other professionals’ input to electric medical records), and consultation support (alternative ordering and alternative schreiber).

Outcomes of the implementation of doctor clerks were categorized into the change in doctor clerk work, change in the perception of physicians, reduction in physician’s time of paperwork, reduction in physician’s overtime work, reduction in hospital cost, reduction in physician’s fatigue, reduction in medical errors, decrease in patient’s waiting time, patient quality of patient care.

Analysis

Each paper was summarized in a table, and the categorized data was descriptively analyzed. Each category of extracted data was analyzed descriptively.

Ethical Consideration

The Unnan City Hospital Clinical Ethics Committee approved the study protocol (No. 20230032).

Results

Selection Flow

**Figure 1:**
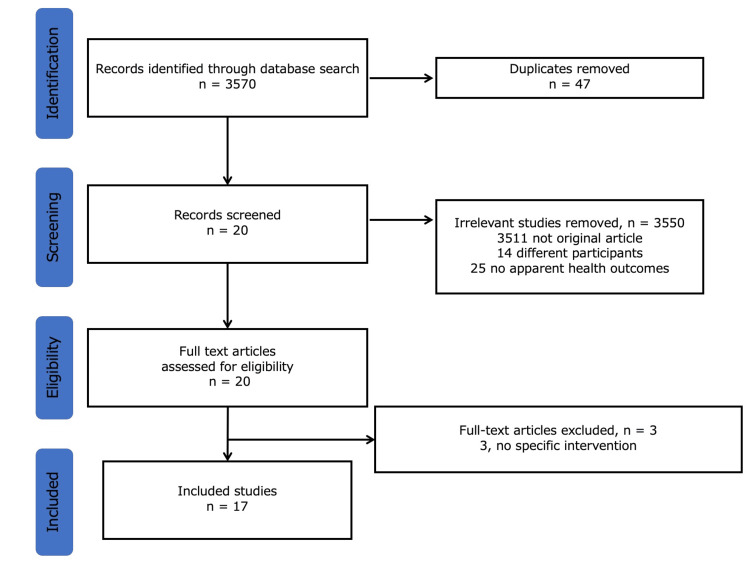
The selection flow (PRISMA diagram) PRISMA: Preferred reporting items for systematic reviews and meta-analyses

Overall, 3570 studies were identified. Of these, 47 duplicate studies were excluded. After reviewing the abstracts, 3550 studies were excluded for the following reasons: 3511 were not original articles; 14 had different participants; and 25 had no apparent health outcomes. Finally, 17 studies were identified in the final analysis after excluding three articles through the eligibility assessment (3, not specific intervention) (Figure 1). 

Each article was categorized into title, settings, department, working conditions, and outcomes. Details on the 17 articles are presented in Table 2.

**Table 2:** The contents of the selected articles

Year	Title	Setting	Department	Working contents	Outcome
2009 [11]	Survey of services provided by medical office assistants and entry of data into electronic health records	General Hospital	Non-specific	Document support, call response, secretary work	Content of doctor clerk works
2011 [12]	Effect of new department of physician's assistance in hospital: from paper works to healthcare quality improvement	General Hospital	Non-specific	Document support, education support, research support, conference support	Perception of physicians
2011 [13]	Improvement in clerical work of physicians through the introduction of medical administrative staff	General Hospital	Orthopedics	Document support	Reduction in time of paperwork, reduction in overtime work, reduction in hospital cost
2012 [14]	The survey of the work sharing among physician assistants and healthcare professions in 23 hospitals in Chugoku-Sikoku block of the national hospital organization	General Hospital	Non-specific	Document support, consultation support, other professional support	The difference in additional care in the implementation of doctor clerks
2013 [15]	Standardization of the work of the “doctor's office work assistant (medical clerks)”, and management by the log data of the hospital information system	University Hospital	Non-specific	Document support	Doctor clerks' education for standardization
2014 [16]	The analysis of improvement in clerical work of orthopedic surgeons through the introduction of doctor assistant staff	General Hospital	Orthopedics	Document support	Reduction in time of paperwork, reduction in overtime work, reduction in hospital cost
2014 [17]	The effect of discharge summary creation support utilizing medical assistants	General Hospital	Non-specific	Document support	Perception of physicians
2016 [18]	Doctors' present evaluation on medical assistant clerk and secretaries’ future duties	General Hospital	Non-specific	Document support	Content of doctor clerk works
2017 [19]	Current status of the doctor’s clerk in Shiga prefecture	General Hospital	Non-specific	Document support, consultation support	Content of doctor clerk works
2018 [20]	Evaluation of usefulness of medical assistance to part-time doctors	General Hospital	Pulmonology	Consultation support	Reduction in fatigue of part-time physicians
2019 [21]	The use of doctor clerks and its effects in critical care and emergency centers	University Hospital	Non-specific	Document support, consultation support	Reduction in time of paperwork, reduction in overtime work, increase in the number of acceptance of patients
2019 [22]	Benefit of doctors' assistant on medical safety	University Hospital	Emergency	Document support	Reduction in medical incidents
2019 [23]	The analysis of the effect of doctor assistants on hospital management in the balance of the receipts against expense	General Hospital	Orthopedics	Document support	Cost of hospitals
2020 [24]	To perform outpatient and ward work smoothly: improvement of work efficacy by introducing medical assistants	General Hospital	Neurosurgery	Document support, consultation support, ordering support	Reduction in time of paperwork, perception of physicians
2020 [25]	Task of scribes to improve physician's work environment and reduce heavy workload: results from questionnaire for physicians and scribes	General Hospital	Non-specific	Document support, consultation support, ordering support	Reduction in time of paperwork, reduction in overtime work, decrease in patient waiting time
2020 [26]	The usefulness of medical clerk who support doctor's office work in our emergency medical center	General Hospital	Emergency	Document support, consultation support, ordering support	Perception of physicians
2022 [27]	Doctor clerk implementation in rural community hospitals for effective task shifting of doctors: a grounded theory approach	Community Hospital	General Medicine	Document support, consultation support, ordering support	Primary to tertial prevention

Clinical Settings

Based on the review, most studies were performed in general hospitals with 76.5% (13/17). The studies regarding university hospitals were 17.6% (3/17). Only one study was performed in a community hospital with 5.9% (1/17).

Charge Department

The review shows that more than half of doctor clerks worked not explicitly allocated to one department and did their work not specific to one department with 52.9% (9/17). Three studies report that doctor clerks collaborate with orthopedic surgeons. Two studies report that doctor clerks collaborate with emergency medicine physicians. Each study reports that doctor clerks collaborate with respiratory or general medicine.

Working Contents

Regarding working content, the most frequent is document support, with 94.1% (16/17). The second most frequent working content is consultation support, with 47.1% (8/17). The third most frequently working content is ordering support, with 23.5% (4/17). Call response, secretary work, education support, research support, conference support, and other professional support are included, each with 5.9% (1/17).

Clinical Outcomes

Regarding clinical outcomes, five studies assessed a reduction in physician paperwork time (29.4%). Four studies assessed the frequency of the contents of doctor clerks’ work (23.5%). Four studies assessed the positive perception of physicians (23.5%). Four studies assessed the amount of the reduction in physicians’ overtime work (23.5%). Three studies assess the amount of the reduction in hospital costs (17.6%). One study assessed part-time physicians' fatigue reduction (5.9%). Each study assessed the quality of patient care, such as doctor's clerk education for standardization, increase in the number of patients accepted, reduction in medical incidents, decrease in patient waiting time, and primary to tertiary prevention.

Discussion

This paper's systematic review highlights doctor clerks' emerging role in Japan's healthcare system, illustrating a significant shift towards optimizing physicians' work environment. The introduction of doctor clerks, primarily in general hospitals, reflects an innovative approach to addressing the dual challenges of clinical care and administrative duties.

Regarding effectiveness in task shifting and workload reduction of physicians, the predominance of document support as the primary task among doctor clerks (94.1%) aligns with the initial intent of their role to alleviate the administrative burden on physicians [28]. This task shifting is evident in the reduced paperwork time and physician overtime work, as reported in several studies [18,21,27]. Expanding roles to include consultation and ordering support also signifies the evolving nature of the doctor clerk's responsibilities, potentially leading to a more holistic approach to assisting physicians [26,27]. Doctor clerks can be involved in health promotion, vaccination, and primary to tertiary prevention for better quality patient care [27]. To promote their effective working of quality of patient care, doctor clerks should participate in the discussion of quality improvement of patient care.

Regarding perception and satisfaction among physicians, the positive perception of physicians towards the assistance doctor clerks provide is noteworthy, but collaboration with family medicine should be promoted for healthcare. This satisfaction can be attributed to the reduced administrative workload, allowing physicians to focus more on patient care [2,21,26]. However, it is essential to balance the expanded roles of doctor clerks with the need to maintain the quality of administrative tasks, ensuring that the shift in responsibilities does not lead to a decline in the quality of these tasks [18,19]. In addition, in community hospitals, the collaboration among doctor clerks and family physicians is essential for better patient care because family physicians are the main stakeholders in interprofessional collaboration for health prevention and promotion [27,29]. For enhanced evidence to clarify the effectiveness of doctor clerks, the collaboration between family physicians and doctor clerks should be investigated in the literature.

Regarding the impact on patient care and hospital operations, while there is evidence of operational benefits such as reduced hospital costs, the direct impact on patient care quality remains unclear. Studies mentioning decreased patient waiting times and reduced medical incidents are promising, but this area requires more focused research [27,29]. The study assessing the role of doctor clerks in primary to tertiary prevention in a community hospital setting offers a unique perspective on their potential impact on patient care [27,29]. The lack of health outcome evidence can be supplemented by the collaboration with family physicians dealing with health promotions and prevention in primary to tertiary care, especially in rural contexts [30]. 

This systematic review, while comprehensive, has several limitations. Firstly, the review is constrained by language and region, focusing exclusively on studies conducted in Japan and available in English or Japanese. This geographic and linguistic restriction may limit the generalizability of the findings to other contexts. Secondly, the exclusion of non-empirical studies, such as editorials and news articles, may have omitted valuable insights and perspectives on the role and impact of doctor clerks. Thirdly, the reliance on specific search terms may have inadvertently excluded relevant studies not using these terms. Lastly, the potential for publication bias cannot be discounted, as studies reporting positive outcomes may be more likely to be published, skewing the overall understanding of the effectiveness and impact of doctor clerks.

## Conclusions

Introducing doctor clerks in Japan's healthcare system is a promising step towards alleviating the administrative burden on physicians, potentially leading to improved patient care. While the initial findings are encouraging, particularly regarding operational efficiency and physician satisfaction, further research is needed to determine the impact on patient care quality conclusively. This study is a foundation for future investigations and policy considerations in healthcare system optimization.
